# Direct evidence of sex and a hypothesis about meiosis in Symbiodiniaceae

**DOI:** 10.1038/s41598-021-98148-9

**Published:** 2021-09-22

**Authors:** R. I. Figueroa, L. I. Howe-Kerr, A. M. S. Correa

**Affiliations:** 1 Spanish Institute of Oceanography in Vigo (IEO-CSIC), Subida a Radio Faro, 50, 36390 Vigo, Spain; 2 BioSciences Department, Rice University, Houston, TX USA

**Keywords:** Cell biology, Cell division, Meiosis, Ocean sciences, Marine biology

## Abstract

Dinoflagellates in the family Symbiodiniaceae are obligate endosymbionts of diverse marine invertebrates, including corals, and impact the capacity of their hosts to respond to climate change-driven ocean warming. Understanding the conditions under which increased genetic variation in Symbiodiniaceae arises via sexual recombination can support efforts to evolve thermal tolerance in these symbionts and ultimately mitigate coral bleaching, the breakdown of the coral-Symbiodiniaceae partnership under stress. However, direct observations of meiosis in Symbiodiniaceae have not been reported, despite various lines of indirect evidence that it occurs. We present the first cytological evidence of sex in Symbiodiniaceae based on nuclear DNA content and morphology using Image Flow Cytometry, Cell Sorting and Confocal Microscopy. We show the Symbiodiniaceae species, *Cladocopium latusorum*, undergoes gamete conjugation, zygote formation, and meiosis within a dominant reef-building coral in situ. On average, sex was detected in 1.5% of the cells analyzed (N = 10,000–40,000 cells observed per sample in a total of 20 samples obtained from 3 *Pocillopora* colonies). We hypothesize that meiosis follows a two-step process described in other dinoflagellates, in which diploid zygotes form dyads during meiosis I, and triads and tetrads as final products of meiosis II. This study sets the stage for investigating environmental triggers of Symbiodiniaceae sexuality and can accelerate the assisted evolution of a key coral symbiont in order to combat reef degradation.

## Introduction

Reef-building corals and other marine invertebrates establish obligate symbioses with a diverse group of dinoflagellates in the family Symbiodiniaceae (reviewed in^1,2^). This symbiosis can be disrupted by environmental stressors including elevated sea surface temperatures (SSTs) and increased UV radiation, resulting in bleaching—the mass loss of Symbiodiniaceae cells and/or chlorophyll from the host—and frequently, host mortality^3,4^. Thermal stress due to anthropogenic climate change is recognized as the leading cause of coral reef degradation^5–7^. Despite this, the onset of coral bleaching from 2007 to 2017 occurred at significantly higher SSTs (+ ~ 0.5 °C) than the preceding decade^8^. This suggests that thermally susceptible genotypes may have adapted and/or declined such that the thermal threshold for bleaching has increased. As reefs continue to experience thermal stress under committed (and likely additional) warming due to climate change, supporting the assisted evolution of thermal tolerance in Symbiodiniaceae^9^ is critical to increasing reef resilience^10^ and contributing to the restoration of ecologically and economically valuable ecosystems^11^.

The most direct mechanism for adaptation to environmental challenges is sex^12,13^. Sexual recombination of parental genotypes during meiosis promotes new (and potentially beneficial) genetic combinations in offspring, the basic prerequisite for evolution via natural selection. Indeed, various field observations and experimental evolution studies across diverse taxa have documented that stressful or novel environments can select for higher levels of sexuality^14^, and microorganisms, including Symbiodiniaceae, are predicted to have a high adaptive capacity in selective environments^15,16^. Meiosis is the hallmark of sex, consisting of two nuclear divisions (karyokinesis) and one simultaneous or two successive cytoplasmic divisions (cytokinesis). Recombination in meiosis ‘mixes’ genetic material from both parents to increase genetic variation in the progeny, in contrast to mitosis—the division typical of ordinary cell growth—where daughter cells have the same number and kind of chromosomes as the parent cell^17^.

**Figure 1:**
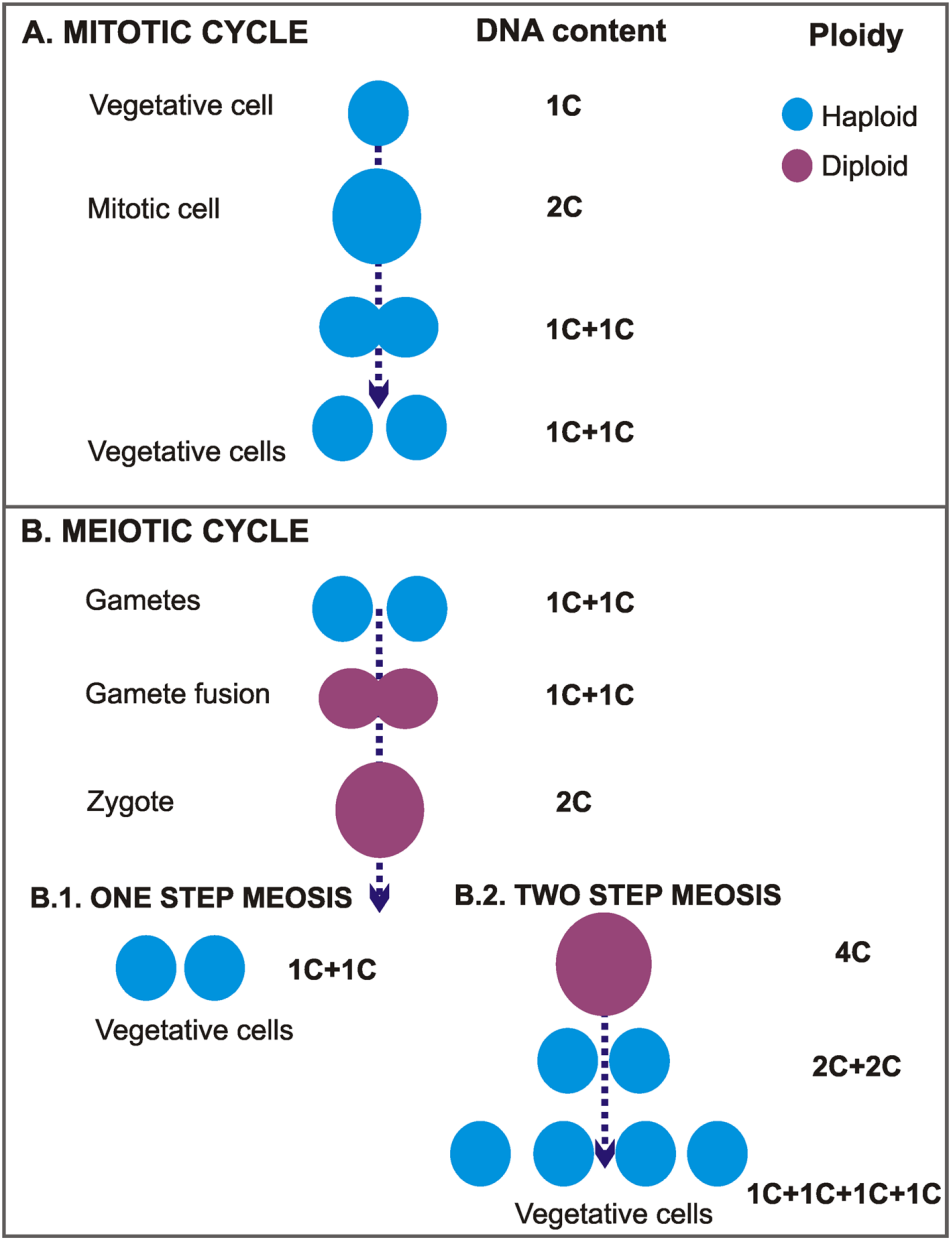
Differences in DNA content and ploidy state between the mitotic and the meiotic cycle, including the two meiotic processes proposed for dinoflagellates (one-step and two-step meiosis).

More than 10% of the approximately 2000 known marine dinoflagellate species produce cysts and are thought to exhibit facultative sexuality during part of their life cycle^18^. In these dinoflagellates, reproduction is primarily asexual (through mitosis, Fig. 1A), but sex can be induced within a subset of cells in a population under certain environmental conditions. Foundational studies, dating back to the 1970s, linked dinoflagellate sexuality to the formation of highly resistant, benthic stages (‘resting cysts’), considered a mechanism for surviving harsh environmental conditions^19^. When resting cysts germinate, meiosis results in the release of novel genotypes that are potentially better adapted to local conditions. Although dinoflagellate sex was first proposed to be rare in nature^20^, research over the last decade revealed that sex in these microeukaryotes is a relatively frequently and flexibly utilized reproductive mechanism. The capacity for sex in dinoflagellates is also now recognized as independent of a species’ ability to form resting cysts (see reviews by^21,22^). Initial studies of *Crypthecodinium cohnii* suggested that dinoflagellates could undergo only a one-step meiosis^23^ (Fig. 1B.1), but later works on different species consistently reported the existence of a two-step meiotic process (^24^ and references therein). In two-step meiosis, there is a delay in meiosis II: a single division occurs in the zygote, whereas the second division takes place at postzygotic stages (Fig. 1B.2). Despite these advances, sexuality remains difficult to identify in most dinoflagellate species due to (i) morphological similarities between sexual and vegetative stages; and (ii) the potential for co-occurrence of 2C DNA content stages derived from both mitosis (haploid) and gamete fusion (diploid) within the same population of cells. Given this, a general consensus has emerged that the detection of a fourfold DNA content stage, which is formed during meiosis (but not mitosis), is key to identifying sex in dinoflagellates^25–29^.

A growing body of molecular evidence shows that Symbiodiniaceae possess functional sexual machinery, and thus suggests that these key coral reef symbionts can reproduce sexually. Indirect evidence for sexual reproduction in this group includes: (i) the existence of a sufficient inventory of essential Symbiodiniaceae meiotic genes^30,31^, as well as genes related to gamete formation^32^; and (ii) population-level genetic signatures^33–36^ and codon usage trends^32^ most parsimoniously interpreted as arising from meiotic recombination. Upregulation of meiosis-related genes has also been documented to occur under thermal stress^37,38^. This temperature-associated regulation suggests that sexual reproduction may be key for the adaptation of Symbiodiniaceae under current warming trajectories, driven by climate change.

In contrast to this growing body of evidence, genomic evidence for the absence of canonical synaptonemal complex (SC), as well as for a reduced set of cohesin complex genes^32^, have been reported from this dinoflagellate family. The synaptonemal complex (SC) mediates the pairing of homologous chromosomes during the early stages of meiotic prophase I and cohesin proteins play a role in sister chromatid cohesion. However, the absence of the SC and reduction of cohesin complex genes does not preclude meiotic capability in Symbiodiniaceae; similar patterns have been reported in other dinoflagellates known to be sexual^32^.

**Figure 2:**
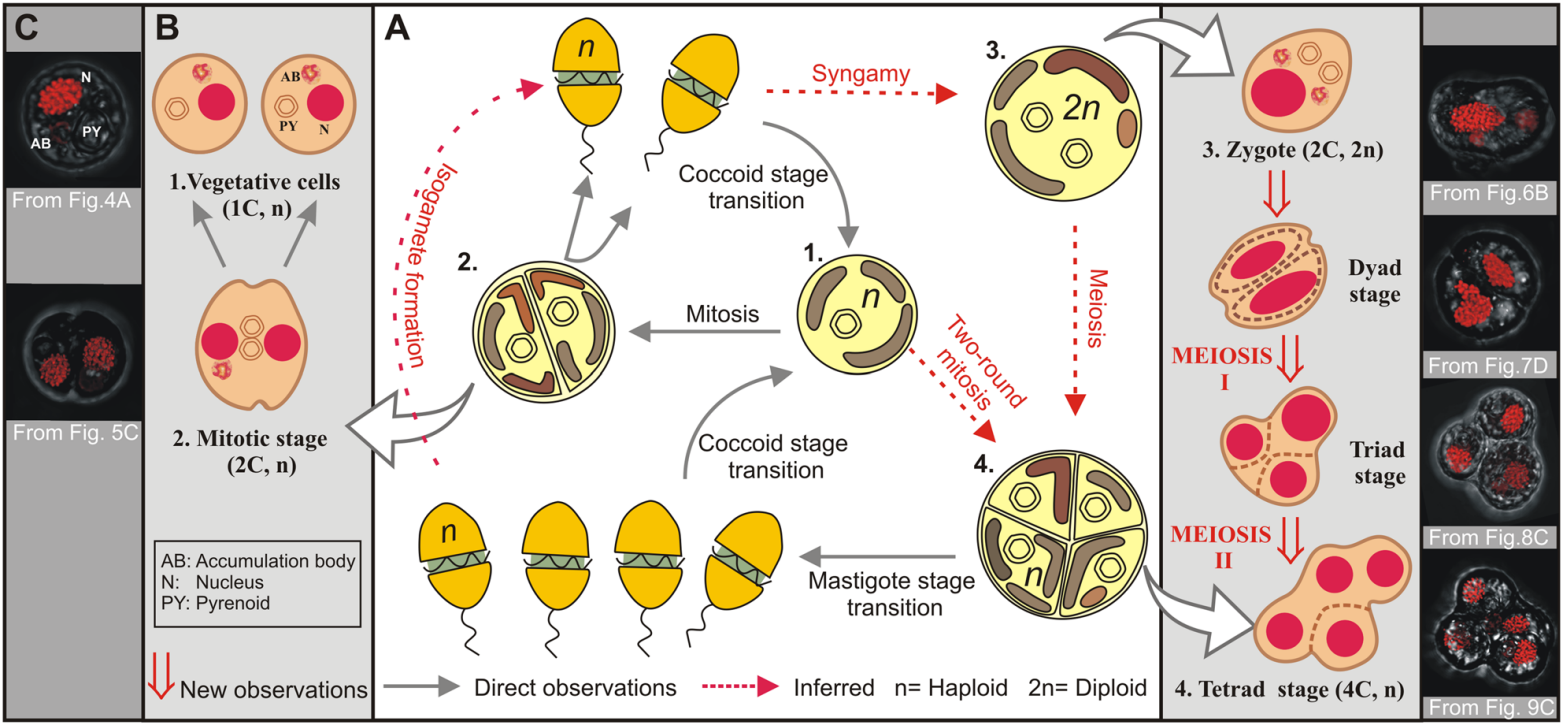
A. Summary of previous direct observations and hypotheses regarding the Symbiodiniaceae life cycle, as well as new information generated by this study. (**A**) Schematic figure (modified from LaJeunesse (http://tolweb.org/), which was based on (Fitt and Trench^40^) summarizing previous direct observations and hypotheses regarding the Symbiodiniaceae life cycle. Previously published direct observations include the production of two mobile haploid cells (mastigotes, referred to as ‘zoospores’ by Freudenthal^39^) from mitosis within the coccoid stage (termed ‘cysts’ or ‘aplanospores’ by Freudenthal^39^), which could behave as isogametes or transform into coccoid stages. The formation of zygotes through gamete fusion, as well as the formation of tetrads (called ‘autospores’ by Freudenthal^39^) via meiosis were hypothesized but not documented. (**B**) Schematic view of the results of the present study in relation to the previously proposed Symbiodiniaceae life cycle (in **A**). Discriminating morphological features (nuclei, pyrenoids and accumulation bodies) are shown in the sexual stages unless in dyads, triads and tetrads, as these stages are transitory and were found in different evolving grades. (**C**) Confocal images corresponding to the proposed sexual stages depicted in (**B**).

Despite strong molecular evidence of sexual reproduction in Symbiodiniaceae, no direct cytological proof for fertilization and meiosis in this group have been available. The first cytological descriptions of Symbiodiniaceae (which was previously recognized as a single genus, *Symbiodinium*^2^) life cycle stages^39^ indicated the existence of motile gymnodinoid zoospores and vegetative cells (the dominant, non-motile stage). Freudenthal^39^ observed that vegetative cells (haploid) either divide by binary fission or form cysts, which are characterized by a thicker wall (Fig. 2A). Cysts could divide or turn into a zoosporangium, which could either release a swimming gymnodinoid zoospore or remain as a non-motile spore (aplanospores, Fig. 2A). In cultures described as “old”, which could indicate nutritional deficiencies, cysts were observed to contain dividing autospores (according to their external morphology, typically two and rarely four). Under certain conditions (not clarified, although the cultures used were clonal), cysts could even give rise to multiple cells resembling a process of gametogenesis. However, morphologies related to gamete conjugation were not detected^39^. Fitt and Trench^40^ subsequently argued that the term “coccoid stage” should be used to describe the non-motile form of Symbiodiniaceae (as opposed to “cyst”). This is because “cyst” in dinoflagellates is usually related to a dormant (non-active), resistant (thick wall) stage, whereas “coccoid stage” can be used independently of a cell’s metabolic activity or cell wall thickness (a highly variable character). Instead, the haploidy of the coccoid (vegetative) stage was considered key to sexuality by Fitt and Trench^40^, who argued that if the coccoid stage was haploid, doublets and emerging motile cells result from a mitotic division, whereas tetrads could represent sexual stages resulting from meiotic division. A summary of this proposed life cycle^41^ is shown in Fig. 2A. Later works based on nuclear reconstructions^42^ and microsatellites^43^ supported this hypothesis of a sexual cycle in Symbiodiniaceae, as they provided molecular evidence of haploidy in vegetative stages of diverse species in *Breviolum* (a Symbiodiniaceae genus formerly known as ‘*Symbiodinium* clade B’^2,43^). Previous work has shown that algal endosymbionts of other dinoflagellate taxa (i.e., *Peridinium balticum*) can sexually reproduce^44^, providing general support for hypothesized sexuality in intracellular symbionts. Although seminal, previous cytological descriptions of the Symbiodiniaceae life cycle and potential sexual stages remain incomplete (e.g., without evidence of gamete fusion) and lack supporting nuclear images and DNA content analyses^39,40^; additional cytological analyses of Symbiodiniaceae life stages are necessary to directly demonstrate sexuality in this key dinoflagellate family of reef symbionts.

Here, we provide new cytological evidence for sexuality in Symbiodiniaceae, focusing on nuclear processes (regardless of motility stage). A combination of flow cytometry techniques (image flow cytometry and sorting) and high-resolution confocal microscope imaging were conducted on populations of the Symbiodiniaceae species, *Cladocopium latusorum*, fixed from the tissues of a dominant coral genus *(Pocillopora* spp.) sampled on a South Pacific reef*.* Our work provides the first direct cytological evidence of meiosis and gamete function in Symbiodiniaceae and suggests that sexual reproduction can occur *in hospite* under natural conditions. These findings open the door to exploring the conditions that promote sex, as well as potential variation in sexual recombination rates, among Symbiodiniaceae species.

## Results

After repeated sampling of three *Pocillopora* coral colonies (containing *Cladocopium latusorum* symbionts) in different thermal and light conditions, 20 samples of preserved Symbiodiniaceae cells (Table 1) were processed and classified into different DNA content groups.

### Image flow cytometry (IFC): some single Symbiodiniaceae cells had higher DNA content than is required for a mitotic division

**Figure 3:**
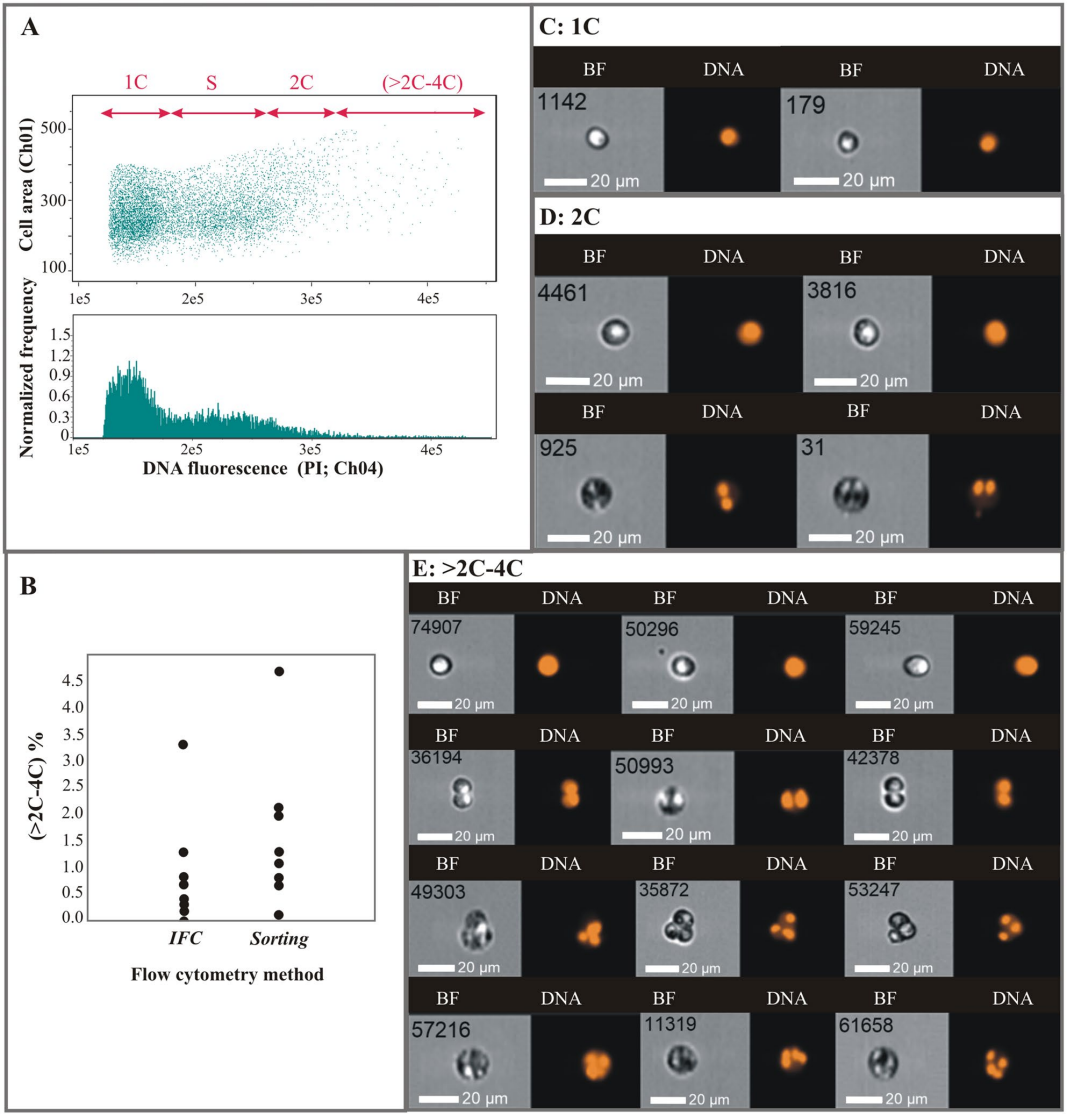
Imaging Flow Cytometry (IFC) results. (**A**) Summary of the categories and delineation process for classifying Symbiodiniaceae cells according to DNA content (representative data from preliminary sample 10 is displayed) and representative images of each DNA content stage (**C**–**E**), where “BF” indicates Bright Field and “DNA” refers to DNA staining. (**B**) Percentage (%) of (> 2C–4C) cells in samples analyzed with IFC versus conventional cytometry (Sorting). (**C**) Cells within the 1C DNA content gate that have a single nucleus. (**D**) Cells within the 2C DNA content gate that have either one nucleus (first row) or two nuclei (second row). (**E**) Cells in the (> 2C–4C) DNA content group, grouped in rows according to the number of nuclei: 1 (first row), 2 (second row), 3 (third row) and 4 (fourth row).

Cells were classified into different DNA content groups according to their propidium iodide (PI) fluorescence from IFC and number of nuclei observed (Fig. 3A). On average, the (> 2C–4C) DNA content group represented a low percentage of samples: typically < 1% of the cells observed (mean of 0.7 ± 1.0) with a maximum of 3.3% of cells per sample (Table 1, Fig. 3B: IFC). “C” DNA content cells had only one roundish nucleus (Fig. 3C), whereas in “2C” DNA content cells, one or two nuclei (Fig. 3D top and bottom rows, respectively) were observed. In “2C–4C” DNA content cells, one, two or three/four nuclei (Fig. 3E-top, middle and bottom rows, respectively) were identified.

**Table 1:** Details of the Symbiodiniaceae samples analyzed in this study and the % of DNA content classifications made from each sample.

Sample ID	Colony ID	Treatment condition	Hours after T0	Time sampled	Analysis type	1C + S	2C	(< 2C–4C)
1	A	C	60	06:00	IFC	92.0	8.0	0
2	A	H	60	06:00	IFC	91.6	7.5	0.8
3	A	C	114	12:00	IFC	94.0	5.3	0.7
4	B	H	60	06:00	IFC	97.5	2.5	0
5	B	C	60	06:00	IFC	96.0	3.8	0.2
6	B	C	114	12:00	IFC	89.8	8.6	0
7	B	H	114	12:00	IFC	82.3	16.4	1.3
8	C	C	60	06:00	IFC	94.8	4.8	0.4
9	C	H	60	06:00	IFC	95.3	4.4	0.3
10	C	C	66	12:00	IFC	60.5	36.2	3.3
11	A	H	78	00:00	Sorting	88.5	7.8	2.0
12	A	H	108	06:00	Sorting	97.9	1.9	0.1
13	A	C	108	06:00	Sorting	89.8	8.4	2.1
14	A	C	114	12:00	Sorting	89.9	9.3	1.1
15	B	H	78	00:00	Sorting	82.9	9.5	4.7
16	B	C	108	06:00	Sorting	86.4	11.5	1.3
17	C	C	108	06:00	Sorting	94.2	4.9	0.8
18	C	H	78	00:00	Sorting	91.9	7.4	1.3
19	C	H	108	06:00	Sorting	96	3.4	0.7
20	C	C	72	18:00	Sorting	91.1	5.9	2.0

All samples analyzed (Sample ID) originated from three colonies of *Pocillopora* species complex (Colony ID) collected from the north shore forereef in Mo’orea, French Polynesia during the dry season (July 2019). Colonies A and B were *Pocillopora meandrina*: Colony C was not identifiable to species. All three colonies contained only *Cladocopium latusorum* symbionts. Treatment Condition: C: Control, ambient temperature, ~ 27 °C; H: Heat stress, ~ 30 °C. ‘Hours after T0’ indicates the cumulative hours that corals had been exposed to the treatment condition at the time of sampling. Analysis Type: IFC = Image Flow Cytometry; Sorting = Sorting Flow Cytometry. A minimum of 10,000 cell nuclei were analyzed for DNA content in each sample.

### Cell sorting and confocal microscopy: clarifying the fine morphology of non-mitotic cells

The percentage of cells in the (> 2C–4C) region based on cell sorting was higher than the IFC results, ranging from 0.1 to 4.7% (mean of 1.61 ± 1.26), although typically below 1.5% (Table 1, Fig. 3B: Sorting). As with the IFC analyses, no significant differences between heat-treated versus control samples, or based on sampling time were found. Sorted cells in each DNA content group were photographed and analyzed morphologically using confocal microscopy as described below.

#### Cells in “1C” DNA content gate

**Figure 4:**
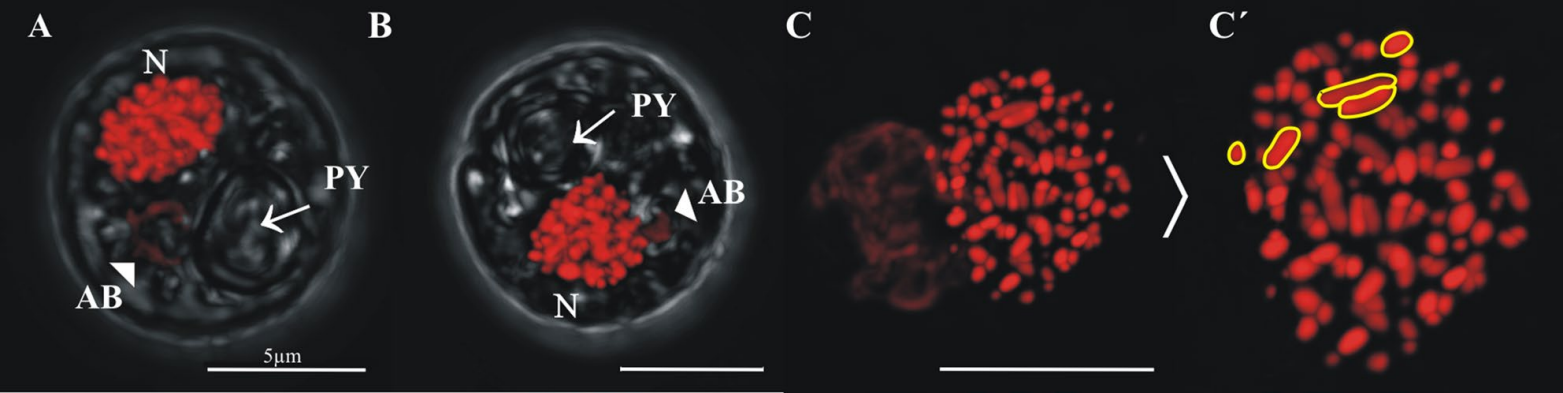
Confocal images of cells sorted in the 1C DNA content gate. (**A**, **B**) representative images of cells showing the pyrenoid (PY, arrow), accumulation body (AB, arrowhead) and nucleus (N). (**C**, **C**′) Images of a nucleus showing individualized chromosomes with varied shapes and sizes (**C**′) examples of individual chromosomes are outlined in yellow).

Cells in the “1C” DNA content region had relatively round outer morphologies, with an ovoid to trapezoid nucleus (N) in which condensed chromosomes were visible; the nucleus was in a peripheral position (Fig. 4). In most “C” cells, a single pyrenoid (PY) was observed as a round depression under transmitted light. Each cell also contained a single accumulation body (AB) that was irregular in size and shape; accumulation bodies were slightly stained by PI (Fig. 4A,B, arrows). Individual chromosomes could sometimes be distinguished (Fig. 4C); chromosome sizes were highly variable (Fig. 4C′).

#### Cells in “2C” DNA content stage

**Figure 5:**
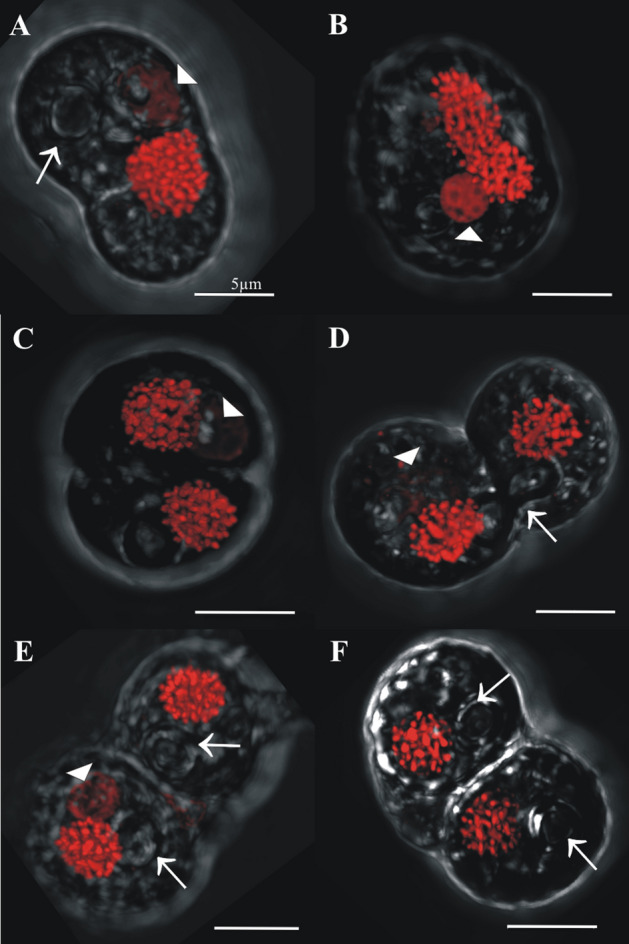
Confocal images of cells sorted in the 2C DNA content gate and classified as part of the mitotic cycle. Arrows denote the pyrenoids and arrowheads denote the accumulation bodies. In these mitotic cells, only one accumulation body/pyrenoid is detected both in single nucleus cells (**A**, **B**) and in pairs which have already duplicated nuclei (**C**). The pyrenoid is shared in advanced mitotic stages (**B**) and is duplicated only during more advanced mitotic phases (**D**–**F**).

**Figure 6:**
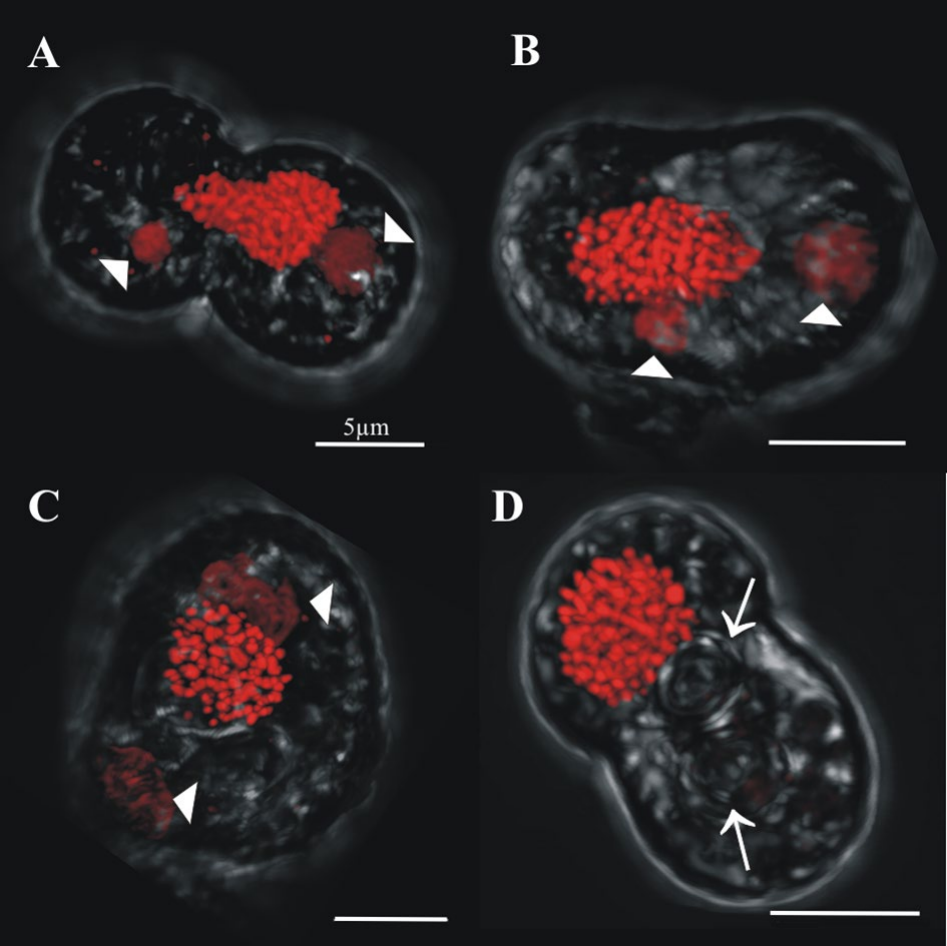
Confocal images of sorted cells in the 2C DNA content gate and with one single nucleus, interpreted as undergoing the meiotic cycle. These cells have duplicated pyrenoids (arrows) and accumulation bodies (arrowheads).

“2C” DNA content cells had either 1 or 2 nuclei. “2C” cells with one nucleus were either classified as part of the mitotic cycle (Fig. 5) or non-mitotic (and therefore, potentially meiotic, Fig. 6) according to the number of pyrenoids and accumulation bodies they contained. Within the mitotic cycle (Fig. 5), cells with 1 nucleus (Fig. 5A,B) varied greatly both in terms of the shape of the nucleus and in the outer morphology of the cell. Most cells had an elongated outer morphology and nucleus, with one pyrenoid (arrow) and one accumulation body (arrow head, Fig. 5A,B). Cells with these characteristics were interpreted as replicating their DNA prior to mitotic division. Progression of mitosis was evident in two-nuclei “2C” cells (e.g., Fig. 5C–F) based on the presence of an equatorial constriction which is formed after nuclear division. As division progresses, the pyrenoid is shared (Fig. 5D, arrow), to later appear clearly in each cell (Fig. 5E–F, arrows), whereas the accumulation body remains single and unshared (Fig. 5E, arrowhead). At the final mitotic stage, nuclei were positioned either opposite or adjacent to each other (Figs. 5E,F, respectively). In the other group of “2C” cells with a single nucleus, classified as non-mitotic cells (Fig. 6), two pyrenoids and accumulation bodies were observed, and the outer morphologies suggested the existence of two newborn cells (splitting apart) or two fusing cells (i.e., mating). Two accumulation bodies (e.g., Fig. 6A–C arrowheads) and two pyrenoids (e.g., Fig. 6D, arrows) were observed in these cells.

#### Cells in (> 2C–4C) DNA content region

**Figure 7:**
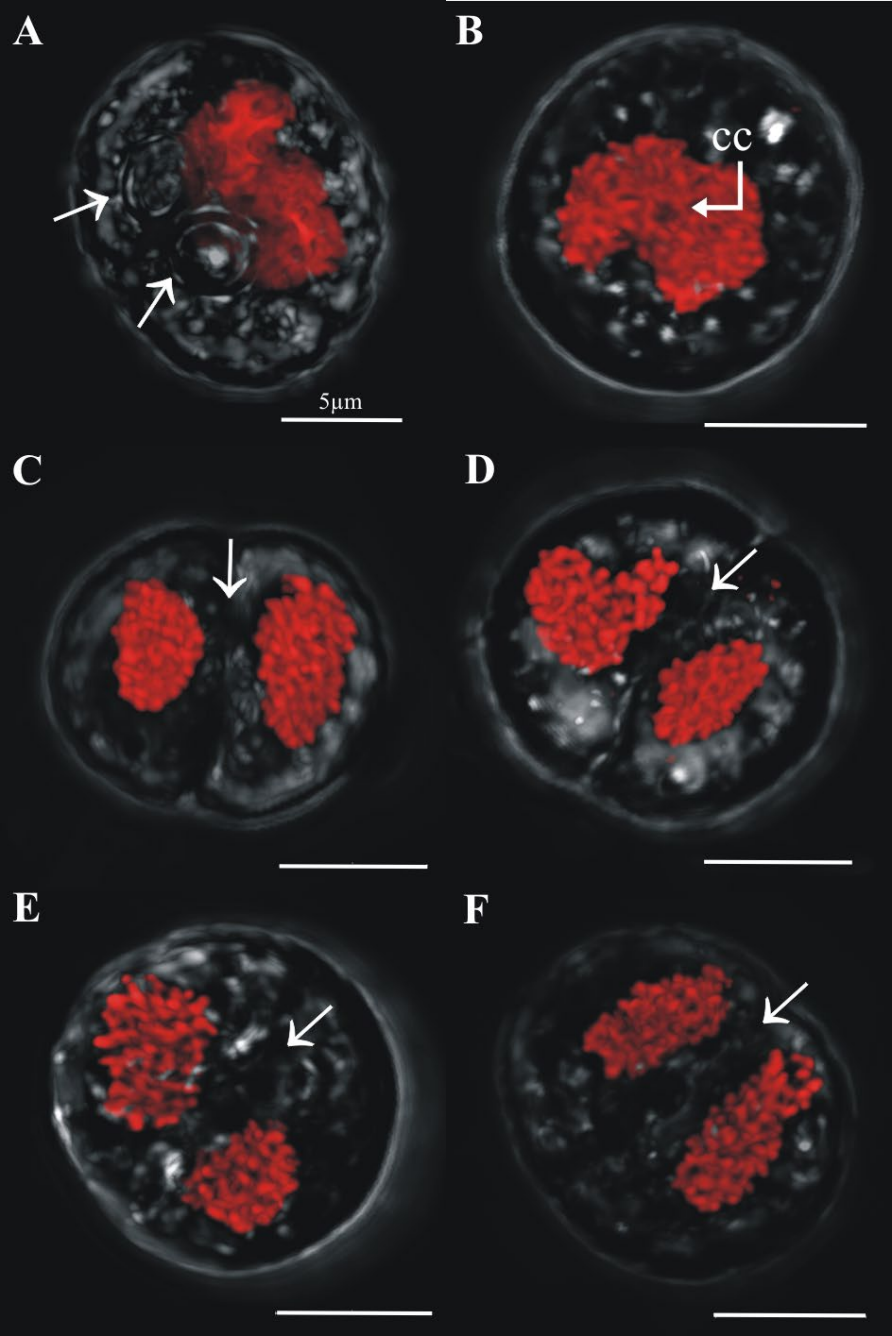
Confocal images of cells sorted in the (> 2C–4C) DNA content gate that have one nucleus or two nuclei. Chromatin was less condensed in these cells, in which two pyrenoids were detected (**A**, arrows). A central cytoplasmic channel (cc, bent arrow) was observed in one of these cells (**B**). Cells with two nuclei (dyads) were considered meiotic and are distinct from two nuclei mitotic cells due to their higher DNA content and the different morphology and shape displayed between both nuclei (**C**–**F**). Dividing furrows in dyads are indicated by an arrow.

**Figure 8:**
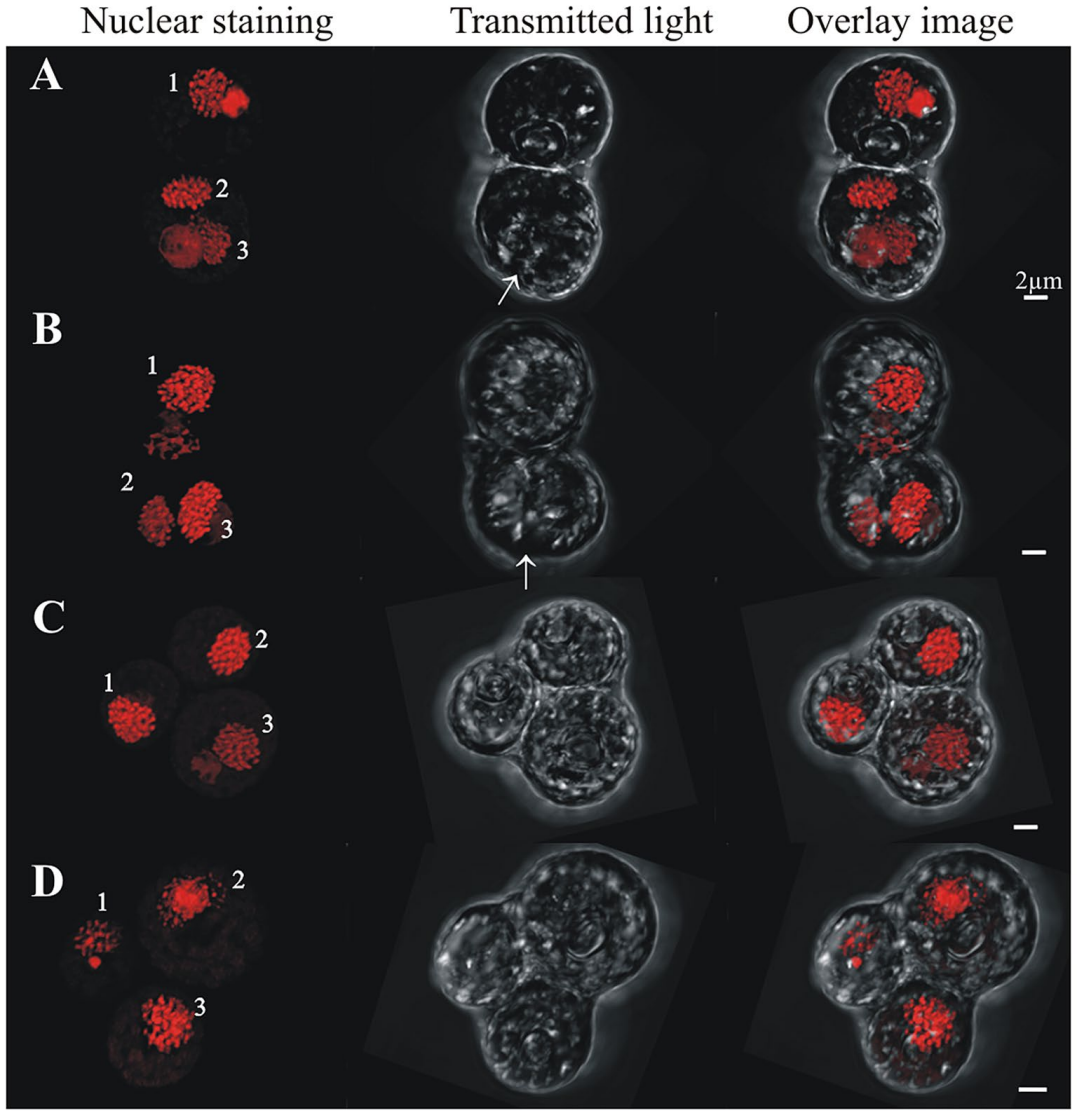
Confocal images of cells sorted in the (> 2C–4C) DNA content gate that have three nuclei and different numbers of lobes in their external morphology (lobes clearly visible in the transmitted light column). (**A**, **B**) cells with two lobes, one showing a dividing furrow (arrow); (**C**, **D**) three lobes.

**Figure 9:**
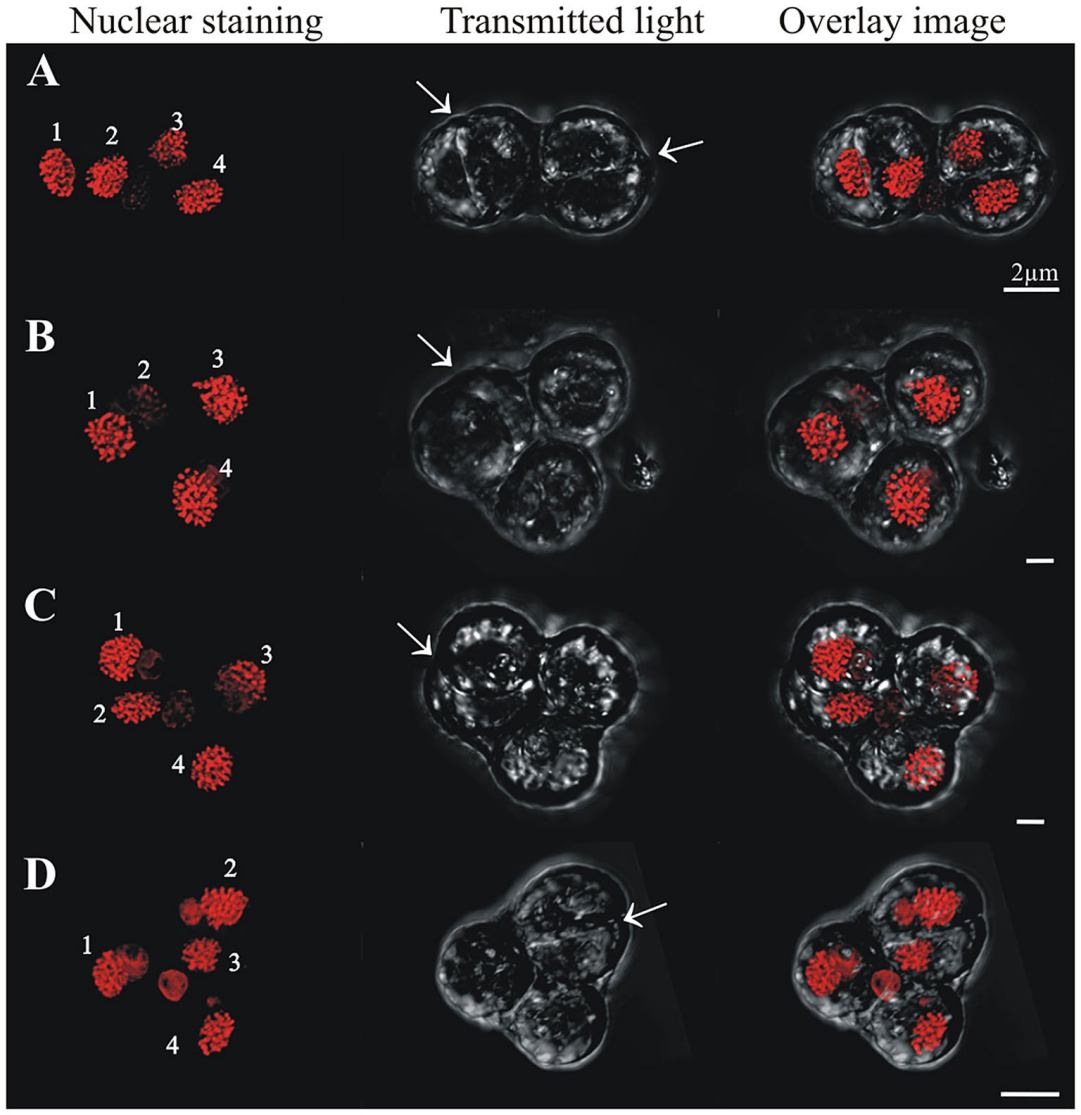
Confocal images of cells sorted in the (> 2C–4C) DNA content gate that have four nuclei and different numbers of lobes in their external morphology (lobes clearly visible in the transmitted light). (**A**) two lobes with a middle furrow of division each (arrows), (**B**–**D**) three lobes with a middle furrow of division in the biggest lobe (arrow).

Cells in (> 2C–4C) DNA content positions could have either one, two, three or four nuclei. In cells with one nucleus, the nucleus was usually irregularly shaped and its chromatin appeared uncondensed (Fig. 7A,B). Two pyrenoids were clear in some cells (e.g., arrowheads in Fig. 7A), and a central nuclear channel (cytoplasmic channel) was occasionally visible (e.g., Fig. 7B arrow). In two-nuclei cells with (> 2C–4C) DNA content, the nuclei often differed from each other in size and shape, although they exhibited the equatorial furrow typical of dividing “2C” DNA content cells (Fig. 7C–F, arrows). In three-nuclei cells (Fig. 8, nuclear staining), which are hereafter termed “triads”, either two lobes (Fig. 8A,B, transmitted light) or three lobes (Fig. 8C,D, transmitted light) were observed in the outer cell morphology. Three-lobed triads were characterized by having one bigger lobe and two smaller ones, whereas in two-lobed triads, one of the lobes was typically undergoing division, as evidenced by the existence of an equatorial furrow within it (Fig. 8A,B, transmitted light, arrows). Four-nuclei cells (Fig. 9), which are hereafter termed “tetrads”, either had two or three lobes in their outer morphology (Fig. 9, transmitted light). For example, Fig. 9A (transmitted image) depicts a tetrad in which only two lobes were observed, and each lobe contained a mid-dividing furrow (arrows). In the tetrads shown in Fig. 9B-D, two of the lobes had no dividing furrow, whereas the third lobe was bigger than the other lobes and displayed a longitudinal furrow separating the two nuclei within (arrow).

#### Time of day, but not temperature or colony ID, influenced Symbiodiniaceae meiotic rates

The percentage of normalized *C. latusorum* cells in the > 2C–4C DNA content category detected with both ImageFlow cytometry and sorting methodologies were compared with the general time of day in which samples were collected: 6 h (time period 1), 12 h (time period 2) and 18–0 h (time period 3). There was a significant effect of the time of the day sampled, with the least sex detected at 6 h and the highest levels of sex found at 18–0 h (Kruskal–Wallis, p = 0.042). No significant differences in the normalized abundances of the (> 2C–4C) DNA content groups were detected between heat-treated versus control samples (Kruskal–Wallis, p = 0.970), or based on colony ID (Kruskal–Wallis, p = 0.947).

## Discussion

Foundational studies previously generated evidence delineating much of the Symbiodiniaceae life cycle, and strong molecular evidence indirectly supported the existence of a sexual cycle in this group of dinoflagellates. However, cytological proof of sexual reproduction in Symbiodiniaceae was still needed to advance our understanding of the basic biology of the ecologically and economically valuable Symbiodiniaceae-coral mutualism, and to catalyze subsequent research into when, where, and how sex occurs in this dinoflagellate group. This study is the first to apply cutting edge approaches (IFC, sorting and confocal analyses) to identify Symbiodiniaceae cells with DNA content and nuclear processes that can definitively be interpreted as sexual activity, including the identification of fusing gametes, zygotes and cells in profase I of meiosis (“4C”, uninuclear cells). Although not a conclusive proof of meiosis, the formation of dyads, triads and tetrads aligns with a meiotic two-step process already described in other dinoflagellates (e.g.^29,45,46^). However, a two-round, asynchronous mitosis cannot be discarded with the available data. Below, we highlight key DNA content and cell morphology observations that allow us to establish differences between mitosis and meiosis in Symbiodiniaceae cells, compare our hypothesis for sex in Symbiodiniaceae to the sexual stages reported in other dinoflagellate species, and highlight outstanding questions regarding the conditions that promote Symbiodiniaceae sex.

### Identifying sexual stages: key differences between mitotic and meiotic cells

Image flow cytometry (IFC) indicated that most of the Symbiodiniaceae cells processed in this study fell into a single group representing the vegetative stage, characterized by low DNA content (“1C”, haploid) and a single, oval to round-ish nucleus. As recently shown in other dinoflagellate species^47,48^, close examination of the haploid cells with confocal microscopy showed that chromosomes were not all identical as previously thought^49^, but in fact, highly variable in size (e.g., Fig. 4C’).

Other single nucleus cells fell between “1C” and “2C” DNA content and were interpreted to be replicating their DNA as part of the mitotic cycle (“S” phase). However, some cells with a single nucleus had a DNA content higher than “2C”; these cells were consistent with a replicating zygote in meiosis I during a two-step meiosis (Fig. 1B.2).

Following a similar dichotomy to the cells with one nucleus, cells with two nuclei could have either “2C” DNA content and be in a mitotic cycle, or have a DNA content > 2C and be in a non-mitotic cycle. The two nuclei of cells in this latter group varied in size, shape and chromatin condensation state; such cells were interpreted as precursors to a triad stage (i.e., cell with three nuclei). To distinguish cells that were part of this non-mitotic sequence, cells with two nuclei and DNA content between “2C” and “4C” were considered dyads, as opposed to what we will simply call “mitotic coccoid stages”. Cells with three and four nuclei (triads and tetrads, respectively) were also detected. Although the morphologies of these stages were difficult to analyze in the IFC images (Fig. 3D,E), such cells could be examined at higher magnification using sorting and confocal microscopy. The variability observed in tetrad morphology (Fig. 9) indicates these cells were dividing stages derived from triads (Fig. 8), in what we interpret to be a delayed meiosis II. Taken together, we infer that the observed Symbiodiniaceae tetrad cells were undergoing two-step meiosis (Fig. 1B.2); this reproductive strategy has been observed in most studied dinoflagellates. For example, *Prorocentrum micans* and *Prorocentrum minimum* form tetrads as a final meiotic product^29^. Additionally, asynchronous divisions of the zygote in these *Prorocentrum* species also lead to the formation of triads^29^. It should be noted that some free-living dinoflagellates (e.g., members of the genus *Alexandrium*) produce chains of cells during two consecutive mitotic divisions. In these *Alexandrium* species, cell chains are formed frequently and are readily detectable in any growing culture. Since Symbiodiniaceae do not form such chains in culture, it is unlikely that the low percentage of tetrad cells observed in our study represent the product of a two-round mitosis. However, this possibility cannot be totally ruled out with the present data. Instead, this study documents Symbiodiniaceae cells with (> 2C–4C) DNA content and a single nucleus that have a morphology consistent with a replicating zygote—such a cell stage is not possible within a mitotic cycle and thus constitutes the first direct proof of meiosis in this dinoflagellate family.

If Symbiodiniaceae engage in sexual reproduction, albeit at low levels within the observed cell populations, then other sexual stages, such as zygotes, should also be observable. Here, we briefly review how mitosis proceeds in Symbiodiniaceae since cells diverging from this process can be recognized as zygotes. According to Freudenthal^39^, during the mitotic division process, all cellular inclusions are equally distributed among the daughter cells with the exception of the accumulation body, which persists as a single unit in the parent cell. Karyokinesis occurs followed by cytoplasmic division, which is initiated by the formation of an equatorial zone of constriction. Following mitotic division, each new daughter cell produces a new cell wall within the old cell wall of the parent cell^50,51^. The old cell wall material is then degraded via an unknown process^52^, releasing the daughter cells. Based on this description of mitosis, only one accumulation body should be present in mitotically dividing cell stages (as shown in Fig. 5). Some of the 2C-single nucleus cells in this study were therefore identifiable as zygotes because they contained two accumulation bodies (Fig. 6A–C); such cells could not have been undergoing mitotic division.

The nuclear morphology and pyrenoid count of some cells also allowed zygotes to be distinguished from mitotically dividing cells. For example, 2C-two nuclei cells with duplicated pyrenoids would only be observed at advanced stages of mitotic division; such cells were frequently detected in our study (e.g., Fig. 5C,D). However, we also observed cells that contained only one “2C” DNA content nucleus but had two well-developed pyrenoids (Figs. 6D, 7A); such cells could not have been undergoing mitosis and were interpreted as fusing gamete products or early stage zygotes (these differences in accumulation bodies and pyrenoids are summarized in Fig. 2B.2 mitotic stage versus 2B.3 zygote).

### Sexual stages of Symbiodiniaceae are similar to those reported in free-living dinoflagellates

**Figure 10:**
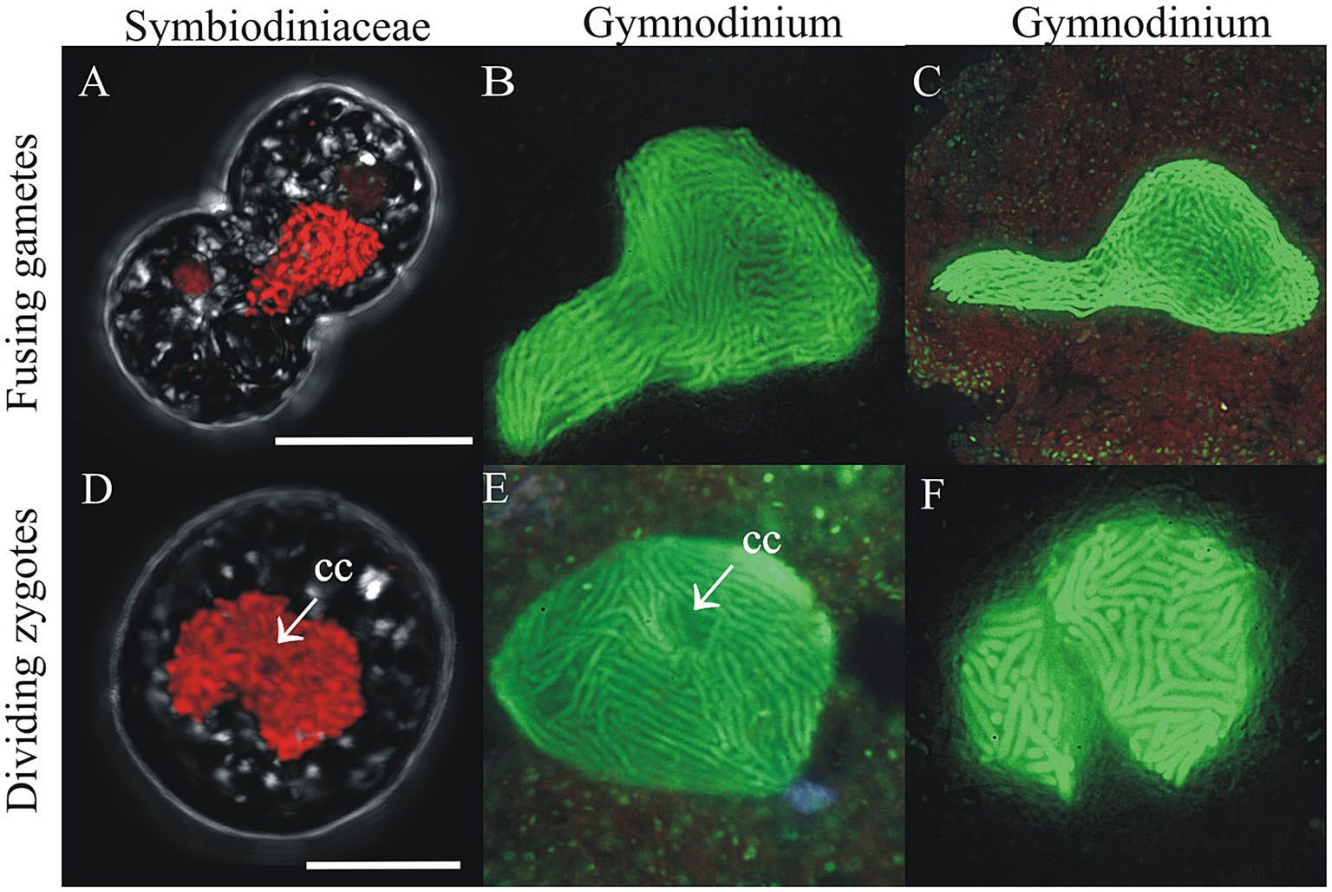
Comparative images show similarities between sexual stages of Gymnodiniaceae and Symbiodiniaceae. (**A**–**C**) *Fusing gametes*. (**A**) Putative gamete fusion in Symbiodiniaceae. (**B**, **C**) Nuclear fusion during syngamy in *Gymnodinium catenatum*. (**D**–**F**) *Zygotes.* (**D**) Putative zygote nucleus in Symbiodiniaceae. (**E**, **F**) Zygote nuclei in *G. catenatum* and *Gymnodinium nolleri* in early meiosis I*.* A central cytoplasmic channel (arrow, cc) is observed in some zygotes, becoming the bilobed nucleus during meiosis I. Gymnodiniaceae pictures are original, corresponding to the same time lapse series published in^45,46^.

In free-living dinoflagellates, the identification of zygotes in non-resting stage cells (planozygotes) has been impeded due to the high morphological similarity between planozygotes and mitotic cells. In past studies, the number of flagella was considered the hallmark of a zygote (although this characteristic is unreliable due to the weak nature of flagella under fixation). Number of flagella is inapplicable to Symbiodiniaceae, however, since this family alternates between mobile and coccoid stages (Fig. 2A), the latter of which lacks flagella. Therefore, the key to morphological discrimination of zygotes in Symbiodiniaceae (and other dinoflagellates in non-resting stages) lies in differentiating features of sex from mitosis, either during nuclear fusion of gametes or the meiotic process. The process of zygote formation we posit for Symbiodiniaceae here is very similar to that observed in other dinoflagellates. For example, during gamete fusion in the naked dinoflagellates *Gymnodinium catenatum* and *Gymnodinium nolleri*, karyogamy occurs first, and, during the process, one gamete nucleus migrates to the position of the other, and they fuse. This occurs while the cell wall is in early stages of fusion, allowing two fusing cytoplasms to be distinguished^45,46^ (Fig. 10, first row). In other species, such as *Prorocentrum micans*, the process looks similar at the nuclear level, but the cytoplasms never fuse, and instead, one of them degenerates^26^. Early stages of meiosis in Gymnodiniaceae are characterized by a big and round-ish zygotic nucleus that changes into a bi-lobed form with a central ‘cytoplasmic channel’, and a DNA-decondensed state in which chromosomes appear thinner (Fig. 10, second row). In dinoflagellates, chromosome segregation occurs via binding to the nuclear envelope surrounding the cytoplasmic channels and microtubule bundles^53^. Although the formation of cytoplasmic channels was first described during mitosis^54,55^, it was later also confirmed to occur during meiosis. Specifically, a main channel centrally positioned during meiosis I is often visible via conventional fluorescence microscopy^45,46^. Given this, some of the one-nucleus Symbiodiniaceae cells with (2C–4C) DNA content recorded here (Fig. 7A,B) could represent zygotes in early meiosis I.

Some characteristics and processes described here for Symbiodiniaceae have also been reported for members of the plant kingdom. For example, the final product of male meiosis in flowering plants is a tetrad of haploid microspores enclosed in a polysaccharide cell wall, and meiosis I often leads to the formation of dyads. Each dyad can divide again to form tetrads through an asynchronous meiosis II division^56^. In *Arabidopsis*, two nuclear divisions occur before simultaneous cytokinesis yields a tetrad of haploid cells. Additionally, in some *Arabidopsis* mutants, cell divisions are delayed, resulting in the formation of abnormal intermediates, most frequently dyad meiotic products^57^.

**Figure 11:**
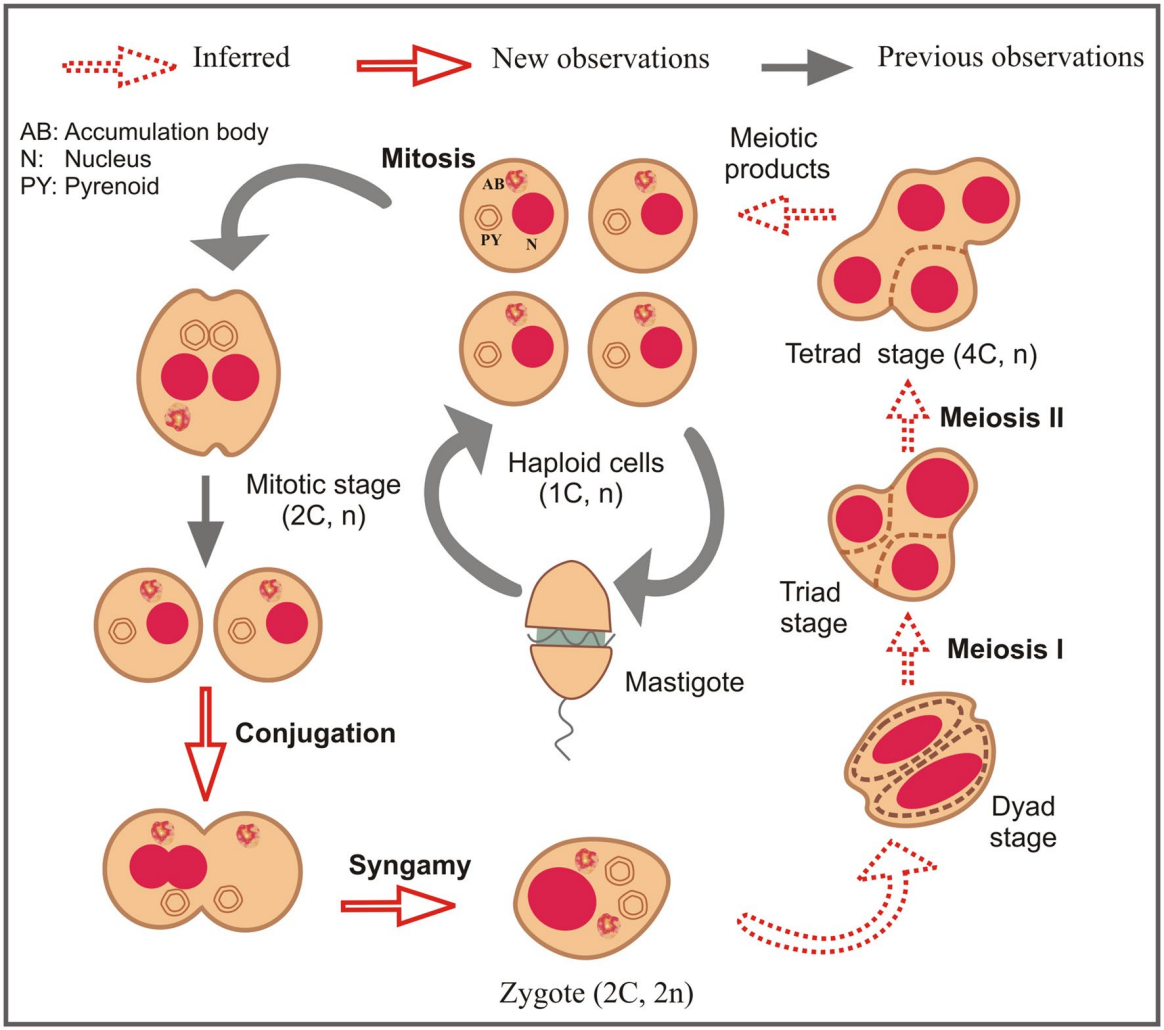
Updated proposed life cycle hypothesis for Symbiodiniaceae, based on the findings of this study. Vegetative cells are characterized by the presence of three unique elements: nucleus (N), pyrenoid (PY) and accumulation body (AB). Mitotic cells replicate their DNA, forming a larger (2C) nucleus. Nuclear division (karyokinesis) occurs first, followed by cytoplasmic division (cytokinesis). Pyrenoids are observed to duplicate in advanced cytokinesis stages, when the outer cellular morphology is already indicative of two cells. Zygotes (2 N, diploid) have a larger nucleus than vegetative cells due to the nuclear fusion of gametes. Two pyrenoids and accumulation bodies are present after fusion as a result of the cytoplasmic contribution of each gamete. DNA replicates once, giving rise to a 4C DNA content cell, which then divides into two cells (dyad) during meiosis I. Asynchronous division during meiosis II leads to the formation of triads and eventually to a 4-cell stage (tetrad) of haploid, 1C DNA content cells.

The hypothesized process for gamete conjugation proposed here aligns with previous observations of Symbiodiniaceae by Freudenthal^39^ and Fitt and Trench^40^. These researchers indicated that during Symbiodiniaceae cell division, karyokinesis (nuclear division) occurs first, and later an equatorial zone of constriction in the cytoplasm separates the two daughter cells, which split pyrenoids but not accumulation bodies. As occurs in other dinoflagellate species (e.g.^45,46^), we propose that nuclear fusion is faster than cytoplasmic fusion during the process of gamete conjugation, given the existence of cells with elongated external shapes and duplicated pyrenoids and accumulation bodies but a single “2C” DNA content nucleus; such cells further corroborate ongoing gametogenesis and zygote formation. Thus, the hypothesized Symbiodiniaceae life cycle (summarized in Fig. 2A) put forward by initial, foundational works^39,40^ constitutes the foundation of our updated life cycle (Figs. 2B,C, 11). This study documents the entire meiotic process in Symbiodiniaceae and includes: (i) the novel observation of (2C–4C) DNA content cells with a single nucleus and duplicated pyrenoids and accumulation bodies (i.e., direct proof of meiosis); (ii) the identification of previously unpredicted intermediate stages, as dyads and triads; and (iii) the first images of tetrads, which had a relatively linear morphology, compared to the previously described coccoid morphology (Figs. 2B4, 9 vs Fig. 2A4, respectively). Integrating our observations with foundational works, we provide a revised proposed life cycle for Symbiodiniaceae (Fig. 11).

### Outstanding questions regarding Symbiodiniaceae sex

Both techniques (IFC and conventional flow cytometry) applied in this study indicated that sex occurs at relatively low levels in Symbiodiniaceae *in hospite* (typically occurring in less than 1% of cells observed in a sample and at a maximum of ~ 5% of cells); sex was only detectable using high resolution imaging following cell sorting. Conventional flow cytometry has a lower capacity to discriminate cells from other fluorescent particles or aggregates; this is likely why the technique reported a slightly higher percentage of cells with (2C–4C) DNA content than IFC analyses (Table 1, Fig. 3B). Regardless, findings from both IFC and conventional flow cytometry agree with previous molecular analyses that indicated Symbiodiniaceae display a mixed reproductive strategy, which is mainly asexual with occasional to frequent sex^35^. In free-living dinoflagellates, sex also originally appeared to occur rarely, such as in the case of severe nutrient deficiency^20^. However, other studies concluded that sexual reproduction in dinoflagellates is probably more common and flexible in nature than previously thought, but induced under species-specific environmental conditions^21,22^.

**Figure 12:**
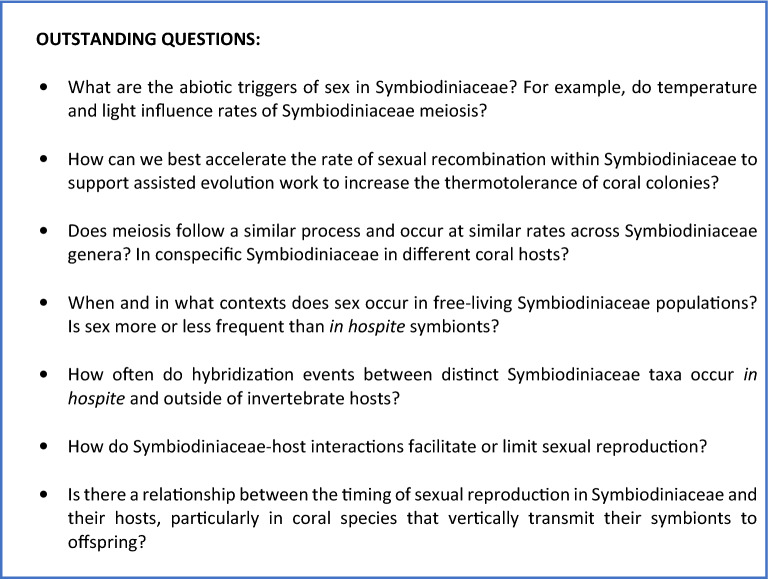
Outstanding question box. Summary of important questions for future research.

Now that sexual stages are confirmed for Symbiodiniaceae, subsequent studies can investigate the biotic and abiotic factors promoting sex, as well as physiological details of Symbiodiniaceae sexual stages. It is worth noting that each colony analyzed in this study contained only *Cladocopium latusorum* symbionts. Rates of sexuality for Symbiodiniaceae *in hospite* may correlate with population- and/or species-level genetic variation; this should be tested in future works. In our study, sexuality occurred more frequently from 18 to 0 h (compared to 6 h and 12 h); this correlation agrees with a previous report for *Alexandrium minutum*^28^, in which sexual 4C peaks were mainly detected during the dark period, after gradually increasing from the final hours of the light period. However, in this study, we did not identify a relationship between sexuality and temperature stress (or colony ID). This is similar to work by Bellantuono et al.^38^, which did not identify enriched GO terms for meiosis I in a *Durusdinium trenchii* strain exposed to elevated versus ambient temperatures, whether the strain was *in hospite* or in culture. Levin et al.^37^, however, reported ≥ fourfold up-regulation of meiosis genes, as well as enrichment of meiosis functional gene groups, in two heterogeneous cultured populations of *Cladocopium* exposed to elevated temperatures. Additional studies that more comprehensively test for the abiotic and biotic triggers of sexuality in a diversity of Symbiodiniaceae strains, populations, species and genera are needed (Fig. 12). Advancing our understanding of the role of Symbiodiniaceae sex in nature, as well as constraints on sex in this group, can potentially be leveraged to enhance the resilience of reef coral colonies to climate change. For example, the induction of new genetic diversity within a Symbiodiniaceae species via enhanced sexuality, may contribute to rapid symbiont adaptation to thermal stress^58^. Indeed, sexual reproduction could underlie local adaptation of Symbiodiniaceae populations to thermal stress, and help explain some within host species variation in bleaching susceptibility^59–61^. Assisted evolution efforts could potentially leverage this by generating more thermally robust populations of homologous symbionts, which can then be provided to aposymbiotic recruits of horizontally transmitting coral species^62,63^.

## Conclusion

This study is the first to categorically demonstrate sexual reproduction in Symbiodiniaceae, establishing a foundation from which to explore the potential role of symbiont evolution in coral resilience to global change. Based on DNA content and morphological evidence, we propose that Symbiodiniaceae species may follow the same two-step meiotic process described for other dinoflagellates, in which the first meiotic division produces a dyad of cells, whereas the second division produces an intermediate triad state, with meiosis II ultimately resulting in a tetrad stage of haplontic cells. This process may be under circadian control, as most putatively meiotic cells were detected at night. Beyond basic biology, understanding sexuality in Symbiodiniaceae can advance experimental evolution work on this group, with the goal of enhancing the capacity of coral holobionts to cope with warming ocean temperatures and other stressors under rapid global change.

## Material and methods

### Symbiodiniaceae sampling and identification

Three morphologically similar colonies of *Pocillopora* species complex^64^ were collected from the forereef on the north shore of Mo’orea, French Polynesia during the dry season (July 2019). To increase the likelihood of sampling distinct genotypes, all sampled colonies were at least 5 m apart from each other. Subsequent restriction fragment length polymorphism (RFLP) digests of mtORF and PocHistone gene amplicons following^65^ confirmed that two sampled colonies (colony ‘A’ and colony ‘B’) were *Pocillopora meandrina*. The third colony (colony ‘C’) could not be identified to species based on^65^ and we therefore refer to it in this manuscript as *Pocillopora* sp. Each colony was split in half and maintained in flow-through seawater aquaria; half of each colony was subjected to an elevated temperature treatment (~ 3 °C above ambient), while the other half was maintained at ambient temperatures. To maximize the chance of detecting meiotic events, samples were collected and preserved from both the heated and ambient tank intermittently throughout the day and night over a three-day period (Table 1). For each sample, one branch (~ 5 cm^2^) was removed from a given coral colony, airbrushed using 0.22 µm filtered seawater, and homogenized using a FISHERBRAND 150 handheld homogenizer (Waltham, Massachusetts). The homogenate was filtered through 70 µm mesh and fixed to a final concentration of 3% formalin. Fixed cells were concentrated by centrifugation and stored at 4 °C until further processing. Details of the samples analyzed in this study (n = 6 time points and 20 replicates total from 3 coral colonies) are provided in Table 1.

All three colonies contained only *Cladocopium latusorum* symbionts (family Symbiodiniaceae^66^) based on IlUMINA MiSeq of the D1-D2 region of 28S Large Subunit rDNA. PCR reactions were performed using the primers LSU1F_illu and LSU1R_illu^67^ and sequenced with PE300 chemistry at Oregon State University’s Center for Genome Research and Biocomputing (Corvallis, OR). After adaptor removal, samples were processed through the DADA2 pipeline^68^, which generated 116 amplicon sequence variants (ASVs). These initial ASVs were clustered using the LULU algorithm^69^, which merges potentially erroneous ASVs based on sequence similarity and co-occurrence patterns. Running LULU using default parameters (84% similarity, 90% co-occurence) resulted in two final (curated) ASVs, both of which were present in all samples (see github.com/LaurenHK/Symbiodiniaceae_meiosis_MS for pre- and post- LULU ASVs). Based on BLAST (blastn) searches to NCBI’s nr/nt database, one ASV was determined to be non-target amplification of coral DNA and therefore discarded; 9,245–81,351 reads remained per sample. All of these remaining reads belonged to an ASV whose best hit was *Cladocopium latusorum* (NCBI Accession #: MW711731.1, query coverage, 98%; 99.7% identity; e-value ≤ 10^–162^).

### Flow cytometry analyses

*Imaging Flow Cytometry (IFC).* Samples were centrifuged at 7000 × g for 5 min and the pellet resuspended in 2.5 mL of cold methanol, where they were stored for at least 12 h at 4 °C to facilitate pigment extraction. Cells were then washed in PBS (pH 7, Sigma-Aldrich, St. Louis, MO, USA) using the same centrifugation conditions, and the resulting pellet was resuspended in a staining solution consisting of 300 µL of propidium iodide (Sigma-Aldrich, 60 µg·mL^-1^) and 30 µL of RNaseA (Sigma-Aldrich, 100 mg· mL^-1^ in PBS) for at least 2 h in darkness before analysis. Samples were washed in PBS twice right before being run on a Flow Sight image flow cytometer (AMNIS, Seattle, WA, USA), with two lasers, emitting at 488 and 405 nm. The samples were run at low speed and data were acquired until 50,000–70,000 total events were recorded. The software Ideas 6.0 (AMNIS Corporation) was used to analyze DNA fluorescence distributions and the morphologies of cells in the images obtained.

*Image and cell cycle analyses by IFC.* A general template for focused Symbiodiniaceae cells was created, in which unfocused cells and aggregates were eliminated. The gradient Root Mean Square (RMS) feature was used to select focused cells, which have a higher gradient than unfocused cells^70^. Aggregates were eliminated by visually plotting cellular area versus nuclear fluorescence. Each cell-cycle phase was delimited by means of a histogram of propidium iodide (PI) fluorescence using 488 laser excitation and a linear scale: the lowest DNA content coincided with the main peak of the population and was named “1C” (Fig. 3A). A region with the same width as “1C” was centered at 2 × the Geometric mean of the 1C population (based on PI fluorescence) and named “2C” (Fig. 3A). The region in between “1C” and “2C” was termed “S” phase (Fig. 3A); cells in this region were interpreted to be in the DNA synthesis phase. A final region was established that ranged from the end of the “2C” region to the cells with the highest detected DNA fluorescence, which had approximately “4C” mean positions. Additionally, regardless of the number of nuclei they contained, cells were classified as "individual" (i.e., one cell observed) or as "in cell chains" (i.e., more than one cell observed) according to the nuclear-aspect ratio (width vs. height of the mask used to more precisely adjust the area to the U-shaped nucleus) and the cell area. The precision of the aspect ratio adjustment was made manually by studying the acquired images. During data collection, between 40,000–70,000 events were acquired in the gating region selected for the Symbiodiniaceae population.

### Cell sorting

Cells were prepared for sorting using the same fixation and staining protocol described above for IFC analyses. Cells were sorted at low speed and in high purity mode on a SH800Z cell sorter (SONY Biotechnology Inc.) equipped with a 488-nm diode laser. Peaks were analyzed using the SH800Z software, and three regions of DNA content were established as described above in the IFC analyses. The entire sample was sorted to increase the number of rare events following two sorting rounds: First, “C” and “2C” cells were sorted until approximately 5000 events were sorted into the “1C” population (control). The rest of the sample was sorted in a second round separating “2C” from “ > 2C–4C”cells. For statistical analyses, populations with different DNA content regions were analyzed using histograms of PI fluorescence in linear scale using FlowJo 10.7.1 (BD, Becton Dickinson & Company).

### Confocal microscopy

Sorted cells were observed using a confocal LEICA SP8 microscope equipped with 3 laser lines (405, 488 and 552 nm) after mounting the cells on slides using ProLong Gold medium antifade reagent (Invitrogen). The preparations were allowed to rest 1-2 h before observations were performed. Visualization was performed using 488 excitation and bright field (PMT trans) in the transmitted light. To improve spatial analyses and decrease the probability of misinterpretation during visual scoring, all images were extracted from 3D videos; all videos are available as Supplementary Files S1–S6. Imaging was performed at 63 × or 100 × magnification using the super-resolution mode LIGHTNING. Images were optimized for best contrast and brightness, and then analyzed, using LASX software (LEICA Microsystems).

### Statistical analyses

Basic statistics (mean, standard deviation (SD)) and tests for equal means (Kruskal Wallis test) comparing treatment conditions (e.g., time of day, temperature, colony ID) were performed using JASP Team (2020), JASP (Version 0.14.1). All tests were performed with a significance level of *p* value = 0.05. Flow cytometry data (IFC and sorting data) were standardized (Z scores) using the following equation, $Z = \frac{X - \upmu }{{\grave{\rm o} }}$, were X is the observation, µ is the media and ò the standard deviation.

## Glossary

*Accumulation body (or PAS body)*: A spherical body in the cytoplasm of dinoflagellates containing electron-dense, fibrous, and membranous material^71^.

*Facultative sexuality*: The condition in which an organism is capable of reproducing both sexually and asexually.

*Meiosis*: Specialized nuclear division of zygotes (sexual division), which reduces the chromosome complement of reproductive cells, which are subsequently formed, by half. In regular meiosis, DNA replication occurs first, followed by the separation of homologous chromosomes in the first meiotic division (meiosis I), and then sister chromatids are separated in the second division (meiosis II).

*One-step meiosis*: A process in which DNA replication is suppressed prior to the formation of reproductive cells. In this process, only a single division is required (to separate sister chromatids).

*Pyrenoid*: In dinoflagellates, a dense structure formed from proteinaceous granules associated with chloroplasts^72^ and proposed to store proteins. Not all dinoflagellates possess these structures.

*Two-step meiosis*: A process in which reproductive cells are formed through two divisions; the second division is delayed and occurs at post-zygotic stages.

## Supplementary Information

Supplementary Video 1.

Supplementary Video 2.

Supplementary Video 3.

Supplementary Video 4.

Supplementary Video 5.

Supplementary Video 6.

Supplementary Video 7.

Supplementary Video 8.

Supplementary Video 9.

Supplementary Video 10.

Supplementary Video 11.

Supplementary Video 12.

Supplementary Video 13.

Supplementary Video 14.

Supplementary Video 15.

Supplementary Video 16.

Supplementary Video 17.

Supplementary Video 18.

Supplementary Video 19.

Supplementary Video 20.

Supplementary Video 21.

Supplementary Video 22.

Supplementary Video 23.

Supplementary Video 24.

Supplementary Video 25.

Supplementary Video 26.

Supplementary Legends.

## Data Availability

Data supporting the conclusions of this article are included in the article and supplementary files. The datasets analyzed during the current study are available from the corresponding authors upon request.

## References

[1] Baker AC (2003). Flexibility and specificity in coral-algal symbiosis: diversity, ecology, and biogeography of Symbiodinium. Annu. Rev. Ecol. Evol. Syst..

[2] LaJeunesse TC (2018). Systematic revision of Symbiodiniaceae highlights the antiquity and diversity of coral endosymbionts. Curr. Biol..

[3] Glynn PW (1996). Coral reef bleaching: facts, hypotheses and implications. Glob. Change Biol..

[4] Baker AC, Glynn PW, Riegl B (2008). Climate change and coral reef bleaching: An ecological assessment of long-term impacts, recovery trends and future outlook. Estuar. Coast. Shelf Sci..

[5] Hughes TP (2017). Global warming and recurrent mass bleaching of corals. Nature.

[6] Hughes TP (2018). Global warming transforms coral reef assemblages. Nature.

[7] Knowlton N (2001). The future of coral reefs. Proc. Natl. Acad. Sci. U. S. A..

[8] Sully S, Burkepile DE, Donovan MK, Hodgson G, van Woesik R (2019). A global analysis of coral bleaching over the past two decades. Nat. Commun..

[9] Van Oppen MJH, Oliver JK, Putnam HM, Gates RD (2015). Building coral reef resilience through assisted evolution. Proc. Natl. Acad. Sci. U.S.A..

[10] National Academies of Sciences, Engineering, and M. *A Research Review of Interventions to Increase the Persistence and Resilience of Coral Reefs*. *A Research Review of Interventions to Increase the Persistence and Resilience of Coral Reefs* (National Academies Press, 2019). 10.17226/25279.

[11] Duarte CM (2020). Rebuilding marine life. Nature.

[12] Becks L, Agrawal AF (2012). The evolution of sex is favoured during adaptation to new environments. PLoS Biol..

[13] Luijckx P (2017). Higher rates of sex evolve during adaptation to more complex environments. Proc. Natl. Acad. Sci. U. S. A..

[14] Lively CM, Morran LT (2014). The ecology of sexual reproduction. J. Evol. Biol..

[15] Correa AMS, Baker AC (2011). Disaster taxa in microbially mediated metazoans: How endosymbionts and environmental catastrophes influence the adaptive capacity of reef corals. Glob. Change Biol..

[16] Van Oppen MJH, Souter P, Howells EJ, Heyward A, Berkelmans R (2011). Novel genetic diversity through somatic mutations: Fuel for adaptation of reef corals?. Diversity.

[17] Lubischer JL (2007). The cell cycle, principles of control. David O. Morgan. Integr. Comp. Biol..

[18] Bravo I, Figueroa RI (2014). Towards an ecological understanding of dinoflagellate cyst functions. Microorganisms.

[19] Pfiester LA, Anderson DM, Taylor FJR (1987). Dinoflagellate reproduction. The Biology of Dinoflagellates.

[20] Pfiester LA (1989). Dinoflagellate sexuality. Int. Rev. Cytol..

[21] Kremp A, Lewis JM, Marret F, Bradley LR (2017). Diversity of dinoflagellate life cycles: Facets and implications of complex strategies. Biological and Geological Perspectives of Dinoflagellates.

[22] Figueroa RI, Estrada M, Garcés E (2018). Life histories of microalgal species causing harmful blooms: Haploids, diploids and the relevance of benthic stages. Harmful Algae.

[23] Himes M, Beam CA (1975). Genetic analysis in the dinoflagellate *Crypthecodinium* (Gyrodinium) *cohnii*: Evidence for unusual meiosis. Proc. Natl. Acad. Sci. U. S. A..

[24] Tillmann U, Hoppenrath M (2013). Life Cycle of the pseudocolonial dinoflagellate *Polykrikos kofoidii* (Gymnodiniales, Dinoflagellata). J. Phycol..

[25] Bhaud Y, Soyer-Gobillard M-O, Salmon JM (1988). Transmission of gametic nuclei through a fertilization tube during mating in a primitive dinoflagellate, *Prorocentrum micans* Ehr. J. Cell Sci..

[26] Soyer-Gobillard M-O, Bhaud Y, Hilaire S (2002). New data on mating in an autotrophic dinoflagellate, *Prorocentrum micans* Ehrenberg. Vie Milieu.

[27] Brosnahan ML (2014). Complexities of bloom dynamics in the toxic dinoflagellate *Alexandrium fundyense* revealed through DNA measurements by imaging flow cytometry coupled with species-specific rRNA probes. Deep Res. Part II Top. Stud. Oceanogr..

[28] Figueroa RI, Dapena C, Bravo I, Cuadrado A (2015). The hidden sexuality of *Alexandrium minutum*: An example of overlooked sex in dinoflagellates. PLoS ONE.

[29] Berdieva M, Kalinina V, Lomert E, Knyazev N, Skarlato S (2020). Life cycle stages and evidence of sexual reproduction in the marine dinoflagellate *Prorocentrum minimum* (dinophyceae, prorocentrales). J. Phycol..

[30] Chi J, Parrow MW, Dunthorn M (2014). Cryptic sex in Symbiodinium (alveolata, dinoflagellata) is supported by an inventory of meiotic genes. J. Eukaryot. Microbiol..

[31] Liu H (2018). Symbiodinium genomes reveal adaptive evolution of functions related to coral-dinoflagellate symbiosis. Commun. Biol..

[32] Shah S, Chen Y, Bhattacharya D, Chan CX (2020). Sex in Symbiodiniaceae dinoflagellates: genomic evidence for independent loss of the canonical synaptonemal complex. Sci. Rep..

[33] Baillie BK, Belda-Baillie CA, Maruyama T (2000). Conspecificity and indo-Pacific distribution of Symbiodinium genotypes (Dinophyceae) from giant clams. J. Phycol..

[34] LaJeunesse TC (2001). Investigating the biodiversity, ecology, and phylogeny of endosymbiotic dinoflagellates in the genus Symbiodinium using the ITS region: In search of a ‘species’ level marker. J. Phycol..

[35] Thornhill DJ, Howells EJ, Wham DC, Steury TD, Santos SR (2017). Population genetics of reef coral endosymbionts (Symbiodinium, Dinophyceae). Mol. Ecol..

[36] Pettay DT, Wham DC, Pinzón JH, LaJeunesse TC (2011). Genotypic diversity and spatial-temporal distribution of Symbiodinium clones in an abundant reef coral. Mol. Ecol..

[37] Levin RA (2016). Sex, scavengers, and chaperones: Transcriptome secrets of divergent symbiodinium thermal tolerances. Mol. Biol. Evol..

[38] Bellantuono AJ, Dougan KE, Granados-Cifuentes C, Rodriguez-Lanetty M (2019). Free-living and symbiotic lifestyles of a thermotolerant coral endosymbiont display profoundly distinct transcriptomes under both stable and heat stress conditions. Mol. Ecol..

[39] Freudenthal HD (1962). Symbiodinium gen. nov. and *Symbiodinium microadriaticum* sp. Nov., a Zooxanthella: Taxonomy, life cycle, and morphology. J. Protozool..

[40] Fitt WK, Trench RK (1983). The relation of diel patterns of cell division to diel patterns of motility in the symbiotic dinoflagellate *Symbiodinium microadriaticum* Freudenthal in culture. New Phytol..

[41] LaJeunesse, T. C., Parkinson, J. E. & Trench, R. K. Symbiodinium. *Tree of Life Web Project* Version 04 (2012).

[42] Blank RJ (1987). Cell architecture of the dinoflagellate Symbiodinium sp. inhabiting the Hawaiian stony coral *Montipora verrucosa*. Mar. Biol..

[43] Santos SR, Coffroth MA (2003). Molecular genetic evidence that dinoflagellates belonging to the genus Symbiodinium freudenthal are haploid. Biol. Bull..

[44] Chesnick JM, Cox ER (1987). Synchronized sexuality of an algal symbiont and its dinoflagellate host, *Peridinium balticum* (levander) lemmermann. BioSystems.

[45] Figueroa RI, Bravo I, Garcés E, Ramilo I (2006). Nuclear features and effect of nutrients on *Gymnodinium catenatum* (Dinophyceae) sexual stages. J. Phycol..

[46] Figueroa RI, Rengefors K, Bravo I (2006). Effects of parental factors and meiosis on sexual offspring of *Gymnodinium nolleri* (Dinophyceae). J. Phycol..

[47] Figueroa RI, Cuadrado A, Stüken A, Rodríguez F, Fraga S (2014). Ribosomal DNA organization patterns within the dinoflagellate genus Alexandrium as revealed by FISH: Life cycle and evolutionary implications. Protist.

[48] Cuadrado Á, De Bustos A, Figueroa RI (2019). Chromosomal markers in the genus Karenia: Towards an understanding of the evolution of the chromosomes, life cycle patterns and phylogenetic relationships in dinoflagellates. Sci. Rep..

[49] Rizzo PJ (2003). Those amazing dinoflagellate chromosomes. Cell Res..

[50] Taylor DL (1968). In situ studies on the cytochemistry and ultrastructure of a symbiotic marine dinoflagellate. J. Mar. Biol. Assoc. U. K..

[51] Kevin MJ, Hall WT, McLaughlin JJA, Zahl PA (1969). *Symbiodinium microadriaticum* Freudenthal, a revised taxonomic description, ultrastructure. J. Phycol..

[52] Wakefield TS, Farmer MA, Kempf SC (2000). Revised description of the fine structure of in situ ‘Zooxanthellae’ genus Symbiodinium. Biol. Bull..

[53] Soyer-Gobillard MO, Ausseil J, Géraud ML (1996). Nuclear and cytoplasmic actin in dinoflagellates. Biol. Cell.

[54] Ris H, Kubai DF (1974). An unusual mitotic mechanism in the parasitic protozoan Syndinium sp. J. Cell Biol..

[55] Bhaud Y, Guillebault D, Lennon J, Defacque H, Soyer-Gobillard MO, Moureau H (2000). Morphology and behaviour of dinoflagellate chromosomes during the cell cycle and mitosis. J. Cell Sci..

[56] Harrison CJ, Alvey E, Henderson IR (2010). Meiosis in flowering plants and other green organisms. J. Exp. Bot..

[57] Magnard JL, Yang M, Chen YCS, Leary M, McCormick S (2001). The Arabidopsis gene tardy asynchronous meiosis is required for the normal pace and synchrony of cell division during male meiosis. Plant Physiol..

[58] Wilkinson SP, Fisher PL, Van Oppen MJ, Davy SK (2015). Intra-genomic variation in symbiotic dinoflagellates: Recent divergence or recombination between lineages?. BMC Evol. Biol..

[59] Baums IB, Devlin-Durante MK, Lajeunesse TC (2014). New insights into the dynamics between reef corals and their associated dinoflagellate endosymbionts from population genetic studies. Mol. Ecol..

[60] Brown BE, Dunne RP, Goodson MS, Douglas AE (2002). Experience shapes the susceptibility of a reef coral to bleaching. Coral Reefs.

[61] Baird AH, Bhagooli R, Ralph PJ, Takahashi S (2009). Coral bleaching: The role of the host. Trends Ecol. Evol..

[62] Buerger P (2020). Heat-evolved microalgal symbionts increase coral bleaching tolerance. Sci. Adv..

[63] Chakravarti LJ, Beltran VH, van Oppen MJH (2017). Rapid thermal adaptation in photosymbionts of reef-building corals. Glob. Change Biol..

[64] Gélin P, Postaire B, Fauvelot C, Magalon H (2017). Reevaluating species number, distribution and endemism of the coral genus Pocillopora Lamarck, 1816 using species delimitation methods and microsatellites. Mol. Phylogenet. Evol..

[65] Johnston EC, Forsman ZH, Toonen RJ (2018). A simple molecular technique for distinguishing species reveals frequent misidentification of Hawaiian corals in the genus Pocillopora. PeerJ.

[66] Turnham KE, Wham DC, Sampayo E, LaJeunesse TC (2021). Mutualistic microalgae co-diversify with reef corals that acquire symbionts during egg development. ISME J..

[67] Pochon X, Wecker P, Stat M, Berteaux-Lecellier V, Lecellier G (2019). Towards an in-depth characterization of Symbiodiniaceae in tropical giant clams via metabarcoding of pooled multi-gene amplicons. PeerJ.

[68] Callahan BJ (2016). DADA2: High-resolution sample inference from Illumina amplicon data. Nat. Methods.

[69] Frøslev TG (2017). Algorithm for post-clustering curation of DNA amplicon data yields reliable biodiversity estimates. Nat. Commun..

[70] Marangon I (2012). Intercellular carbon nanotube translocation assessed by flow cytometry imaging. Nano Lett..

[71] Zhou J, Fritz L (1994). The PAS/accumulation bodies in Prorocentrum lima and *Prorocentrum maculosum* (Dinophyceae) dinoflagellate lysosomes. J. Phycol..

[72] Dodge JD, Crawford RM (1971). A fine-structural survey of dinoflagellate pyrenoids and food-reserves. Bot. J. Linn. Soc..

