# Supplementary material for: Direct evidence of sex and a hypothesis about meiosis in Symbiodiniaceae
Source: Sci Rep. 2021 Sep 22;11:18838. doi: 10.1038/s41598-021-98148-9 (PMC8458349; doi:10.1038/s41598-021-98148-9)
Supplement: Supplementary file 27 — Supplementary Legends. [file 41598_2021_98148_MOESM27_ESM.pdf]

**FIGURE LEGENDS OF SUPPLEMENTARY FIGURES S1-S6** OF "DIRECT EVIDENCE OF SEX AND A HYPOTHESIS ABOUT MEIOSIS IN SYMBIODINIACEAE", BY R. I. FIGUEROA, L. HOWE-KERR AND A.M.S. CORREA.

**Figure S1**: 3D videos of cells in figure 4. **S14A**: 3D video of the cell in figure 4A. **S14B**: 3D video of the cell in figure 4B

**Figure S2:** 3D videos of cells in figure 5. **S25A**: 3D video of the cell in figure 5A**. S25B**: 3D video of the cell in figure 5B. **S25C**: video of the cell in figure 5C**. S25D**: video of the cell in figure 5D. **S25E**: video of the cell in figure 5E. **S25F**: video of the cell in figure 5F.

**Figure S3**: 3D videos of cells in figure 6. **S36A**: video of the cell in figure 6A. **S36B**: video of the cell in figure 6B. **S36C**: video of the cell in figure 6C. **S36D**: video of the cell in figure 6D.

**Figure S4:** 3D videos of cells in figure 7. **S47A**: video of the cell in figure 7A. **S47B**: video of the cell in figure 7B. **S47C**: video of the cell in figure 7C. **S47D**: video of the cell in figure 7D. **S47E**: video of the cell in figure 7E. **S47F**: video of the cell in figure 7F.

**Figure S5:** 3D videos of cells in figure 8. **S58A:** video of the cell in figure 8A**. S58B:** video of the cell in figure 8B**. S58C:** video of the cell in figure 8C. **S58D:** video of the cell in figure 8D.

**Figure S6:** 3D videos of cells in figure 9. **S69A:** video of the cell in figure 9A. **S69B**: video of the cell in figure 9B. **S69C:** video of the cell in figure 9C. **S69D:** video of the cell in figure 9D.
